# Impact of Growth Rate on the Protein-mRNA Ratio in Pseudomonas aeruginosa

**DOI:** 10.1128/mbio.03067-22

**Published:** 2022-12-08

**Authors:** Mengshi Zhang, Kelly L. Michie, Daniel M. Cornforth, Stephen K. Dolan, Yifei Wang, Marvin Whiteley

**Affiliations:** a School of Biological Sciences, Georgia Institute of Technology, Atlanta, Georgia, USA; b Emory-Children’s Cystic Fibrosis Center, Center for Microbial Dynamics and Infection, Georgia Institute of Technology, Atlanta, Georgia, USA; c Institute for Data Engineering and Science (IDEaS), Georgia Institute of Technology, Atlanta, Georgia, USA; University of Wisconsin—Madison

**Keywords:** *Pseudomonas aeruginosa*, protein-mRNA ratios, transcriptomics, cystic fibrosis, proteomics, chemostat cultures, protein-to-mRNA ratio

## Abstract

Our understanding of how bacterial pathogens colonize and persist during human infection has been hampered by the limited characterization of bacterial physiology during infection and a research bias toward *in vitro*, fast-growing bacteria. Recent research has begun to address these gaps in knowledge by directly quantifying bacterial mRNA levels during human infection, with the goal of assessing microbial community function at the infection site. However, mRNA levels are not always predictive of protein levels, which are the primary functional units of a cell. Here, we used carefully controlled chemostat experiments to examine the relationship between mRNA and protein levels across four growth rates in the bacterial pathogen Pseudomonas aeruginosa. We found a genome-wide positive correlation between mRNA and protein abundances across all growth rates, with genes required for P. aeruginosa viability having stronger correlations than nonessential genes. We developed a statistical method to identify genes whose mRNA abundances poorly predict protein abundances and calculated an RNA-to-protein (RTP) conversion factor to improve mRNA predictions of protein levels. The application of the RTP conversion factor to publicly available transcriptome data sets was highly robust, enabling the more accurate prediction of P. aeruginosa protein levels across strains and growth conditions. Finally, the RTP conversion factor was applied to P. aeruginosa human cystic fibrosis (CF) infection transcriptomes to provide greater insights into the functionality of this bacterium in the CF lung. This study addresses a critical problem in infection microbiology by providing a framework for enhancing the functional interpretation of bacterial human infection transcriptome data.

## INTRODUCTION

Opportunistic bacterial pathogens such as Pseudomonas aeruginosa are a primary cause of life-threatening infections in health care settings. P. aeruginosa causes both acute and chronic infections (1) that are often difficult to eradicate with conventional antimicrobial therapies (2, 3). A major challenge in understanding the mechanisms controlling the colonization and persistence of bacterial pathogens such as P. aeruginosa is the lack of knowledge regarding their physiology during human infection. In recent studies, we have leveraged the accessibility and high-throughput nature of RNA sequencing (RNA-seq) to profile bacterial function during human infection, providing new insights into bacterial physiology in the human host (4–12). However, as protein, not mRNA, is the primary functional component of a bacterial cell, these transcriptome studies are reliant on the limited assumption that mRNA levels are predictive of protein abundance.

The advent and widespread availability of high-throughput mRNA and protein quantification have allowed investigations of the correlation between mRNA and protein levels across different phylogenetic taxa, including bacteria, plants, and humans (13–27). These studies have revealed a positive correlation between mRNA and protein across kingdoms, with genome-wide Spearman rank correlation coefficients (ρ) ranging from 0.45 to 0.65. However, in eukaryotes, particular subsets of genes have been shown to be more or less predictive of protein levels (18, 28, 29). For example, cell cycle-regulated genes have a weaker correlation in human cells (30), while in Saccharomyces cerevisiae, cell cycle-regulated and nucleolus-localized genes display higher correlations (31). Compared to eukaryotes, investigations of mRNA-protein correlations in bacteria are somewhat limited (14–17, 20, 22, 23, 32). Two previous studies quantified protein and mRNA abundances for P. aeruginosa grown in test tubes, revealing correlation values of 0.64 to 0.65 (32, 33). However, these and other bacterial studies have been limited to a single growth environment and have not explored mRNA-protein correlations of subsets of genes.

Our understanding of the basic aspects of bacterial physiology, including mRNA-protein correlations, is based largely on *in vitro* growth and, thus, is highly biased toward extremely fast-growing bacteria. Not only are high growth rates not generally the norm in the natural environment, but growth rate are also highly dynamic and can vary widely within a population (34–37). Indeed, previous studies revealed that the growth rates of bacteria in the human body during chronic infection can vary widely depending on the nutritional environment of the infection site and the immune response. For example, the doubling time of P. aeruginosa chronically infecting the lungs of people with cystic fibrosis (CF) has been shown to vary from <1 h to >24 h (35), with population averages of between 2.5 and 4.6 h (37). Thus, there is a gap in knowledge as to what extent changes in the growth rate affect basic bacterial physiology, including the correlation of mRNA and protein. Here, we address this knowledge gap by assessing the impact of the growth rate on P. aeruginosa mRNA-protein correlations by performing high-throughput mRNA and protein quantification of bacterial growth at four growth rates in chemostats. We discovered that while the growth rate did not impact the overall correlation of mRNA and protein, there were subsets of genes that exhibited higher correlations at all growth rates. In addition, we developed and employed an RNA-to-protein (RTP) correction factor that allows the more accurate prediction of protein levels of lowly correlated genes, thus increasing the biological utility of human infection transcriptomes from P. aeruginosa and other bacterial pathogens.

## RESULTS

### Growth of P. aeruginosa at four growth rates.

To evaluate how the growth rate impacts mRNA and protein levels, we grew P. aeruginosa strain UCBPP-PA14 (PA14) in carbon-limited chemostats. Chemostats were used as they allow the continuous-culture growth of a planktonic bacterial population at steady state. The generation (doubling) time and bacterial density are precisely controlled, thus providing the opportunity to study the impact of the growth rate on P. aeruginosa mRNA and protein levels in the absence of confounding variables often used to control the growth rate (e.g., different growth media and different temperatures). A defined, minimal medium containing succinate as the sole carbon and energy source was used (38), and bacteria were cultured in chemostats with generation times of 3, 6, 14, and 25 h at a density of 7 × 10^8^ cells/mL. For each generation time, bacterial cells were harvested for transcriptomic and proteomic analyses at steady state.

### Comparison of the transcriptomes and proteomes of fast- and slow-growing P. aeruginosa cultures.

To begin to assess the impact of the growth rate on the P. aeruginosa transcriptome and proteome, we first compared the RNA and protein levels in the fastest (3-h)- and slowest (25-h)-growing chemostat cultures.

**(i) Transcriptome comparison.** Transcripts from 5,894 genes were detected from cells grown at both growth rates (see Data Set S1, sheet 1, in the supplemental material). A total of 2,362 genes were differentially expressed (*P*_adj_ [adjusted *P*] < 0.05) when comparing the lowest and highest growth rates (Data Set S1, sheet 3), indicating that the growth rate has a tremendous impact on P. aeruginosa gene expression. Enrichment analysis of differentially regulated genes using TIGRFAM functional gene annotation database categories (39) revealed no enrichment of any category, indicating a global change in gene expression (by Fisher’s exact test). Using a more stringent cutoff (fold change of ≥4 and *P*_adj_ of <0.05), 343 genes were differentially expressed, with 27 genes being upregulated and 216 being downregulated at the higher growth rate (Data Set S1, sheet 3). Differentially regulated genes included a large number of operons that have been functionally characterized, including three gene clusters encoding components of type VI secretion systems, two of which (H1-T6SS and H2-T6SS) were upregulated and one of which (H3-T6SS) was downregulated at the higher growth rate. Another operon upregulated at the higher growth rate was *pqsABCDE*, which encodes proteins required for the biosynthesis of quinolines that have antimicrobial and quorum signaling activities. Operons downregulated at the higher growth rate include those involved in glycogen biosynthesis, type III secretion, nitrate metabolism, sulfur metabolism, and branched-chain amino acid metabolism. Operons encoding two low-affinity terminal oxidases were also differentially expressed, including the upregulation of the *bo*_3_ quinol oxidase (*cyoABCDE*) and the downregulation of the *aa*_3_ cytochrome *c* oxidase (*coxA*, *coxB*, *PA14_01310*, and *coIII*) at the higher growth rate.

10.1128/mbio.03067-22.7 DATA SET S1 Comparison and overlap of the transcriptomes and proteomes of fast (3-h doubling time)- and slow (25-h doubling time)-growing P. aeruginosa cultures. Download Data Set S1, XLS file, 2.5 MB. Copyright © 2022 Zhang et al. 2022 Zhang et al. https://creativecommons.org/licenses/by/4.0/ This content is distributed under the terms of the Creative Commons Attribution 4.0 International license.

**(ii) Proteome comparison.** A total of 3,903 proteins were identified in cells from both the fastest- and slowest-growing P. aeruginosa chemostat cultures (Data Set S1, sheet 2), with 2,023 of these proteins being differentially produced (*P*_adj_ < 0.05) (Data Set S1, sheet 4). Enrichment analysis of differentially regulated proteins using TIGRFAM categories revealed the enrichment of several “main” categories (39), including protein synthesis and several metabolic categories, as well as the “subrole” categories of ribosomal proteins and tRNA aminoacylation. Overall, most proteins showed a low magnitude of change, with only 391 proteins having a fold change of >2. Of these proteins, 147 were upregulated and 244 were downregulated at the highest growth rate (Data Set S1, sheet 4). Five of the 10 most differentially increased and 6 of the 10 most differentially decreased proteins are hypothetical proteins. As observed in the mRNA comparison (Data Set S1, sheet 3), many of the proteins were encoded by operons that have been functionally characterized (Data Set S1, sheet 4). Proteins with increased levels at the higher growth rate included 55 components of the 30S or 50S ribosomal subunit. Proteins with decreased levels at the higher growth rate included the *aa*_3_ cytochrome *c* oxidase, proteins involved in branched-chain amino acid metabolism, and proteins involved in phenazine biosynthesis. These results reveal that similar to mRNA, changes in the growth rate have a tremendous effect on the P. aeruginosa protein composition.

### Overlap of differentially expressed genes and proteins in fast- and slow-growing P. aeruginosa cultures.

**FIG 1:**
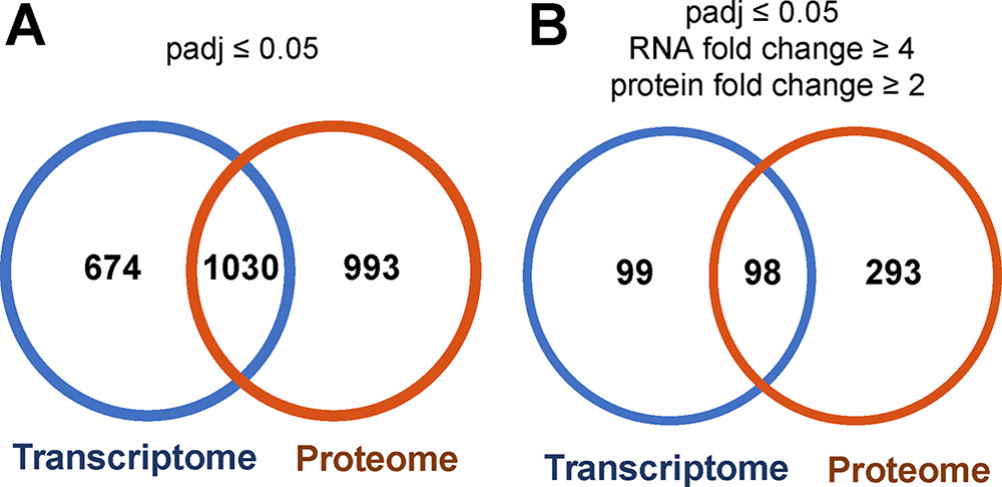
Differentially expressed mRNAs and proteins show modest overlap. Venn diagrams show the overlap between differentially expressed mRNAs and proteins among fast-growing (3-h doubling time) and slow-growing (25-h doubling time) P. aeruginosa cultures. (A) Overlap of differentially produced mRNAs and proteins using a *P*_adj_ value of <0.05. (B) Overlap of differentially produced mRNAs and proteins using a more stringent cutoff (RNA fold change of ≥4, protein fold change of ≥2, and *P*_adj_ of <0.05).

We next asked, of the genes and proteins that are differentially expressed between the highest and lowest growth rates, how many are shared? We focused on the 3,903 genes for which both mRNA and protein were detected at each growth rate. Genes were considered to overlap only if the protein and mRNA levels changed in the same direction (up- or downregulated). Of the 2,697 genes that showed differential mRNA or protein levels (*P*_adj_ < 0.05), 1,030 showed overlapping changes, including 558 that were upregulated and 472 that were downregulated (Fig. 1A, Fig. S1A, and Data Set S1, sheet 5). Enrichment analysis of these shared genes revealed the enrichment of three main TIGRFAM categories: protein synthesis, amino acid synthesis, and purine/pyrimidine/nucleotide/nucleoside synthesis. The use of a more stringent cutoff for differential regulation (RNA fold change of ≥4, protein fold change of ≥2, and *P*_adj_ of <0.05) revealed 98 shared genes between the transcriptomes and proteomes, with 68 genes being downregulated and 30 genes being upregulated (Fig. 1B and Data Set S1, sheet 6). Many of the shared genes were expressed in operons and include the cytochrome *c* oxidase *aa*_3_ (*coxA*, *coxB*, and *PA14_01310*), glycogen biosynthesis (*glgB*, *glgP*, *PA14_36730*, and *PA14_36740*), and sulfur metabolism (*PA14_19540*, *ssuB*, *ssuD*, and *PA14_19590*).

10.1128/mbio.03067-22.1 FIG S1 Assessing the RNA-protein relationships for differentially expressed mRNAs and proteins among fast-growing P. aeruginosa cultures. (A) Scatterplot of mRNA and protein fold changes among fast-growing (3-h doubling time) and slow-growing (25-h doubling time) P. aeruginosa cultures. Genes with differentially expressed (DE) mRNA only (black open circles), protein only (red open circles), and both mRNA and protein (“DE overlap”) (filled blue and pink circles) using the standard *P* value cutoff (*P*_adj_ < 0.05) or a more stringent cutoff (“DE overlap FC”) (mRNA |fold change| of ≥4, protein |fold change| of ≥2, and *P*_adj_ of <0.05) (filled pink circles) are shown. (B and C) Scatterplots of mRNA and protein abundances for genes with differentially expressed mRNA only (gray) or protein only (red) (B) or overlap using either the standard *P* value cutoff (blue) or the more stringent cutoff (pink) (C) for P. aeruginosa grown at the 3-h doubling time. The Spearman rank correlation coefficients between mRNAs and proteins among those gene subsets are 0.50 to 0.64 for ρ_DE_mRNA_only_, 0.55 to 0.62 for ρ_DE_protein_only_, 0.58 to 0.68 for ρ_DE_overlap_, and 0.29 to 0.53 for ρ_DE_overlap_FC_ (*P* < 10^−7^). Download FIG S1, TIF file, 1.1 MB. Copyright © 2022 Zhang et al. 2022 Zhang et al. https://creativecommons.org/licenses/by/4.0/ This content is distributed under the terms of the Creative Commons Attribution 4.0 International license.

### Assessment of mRNA and protein levels across growth rates.

**FIG 2:**
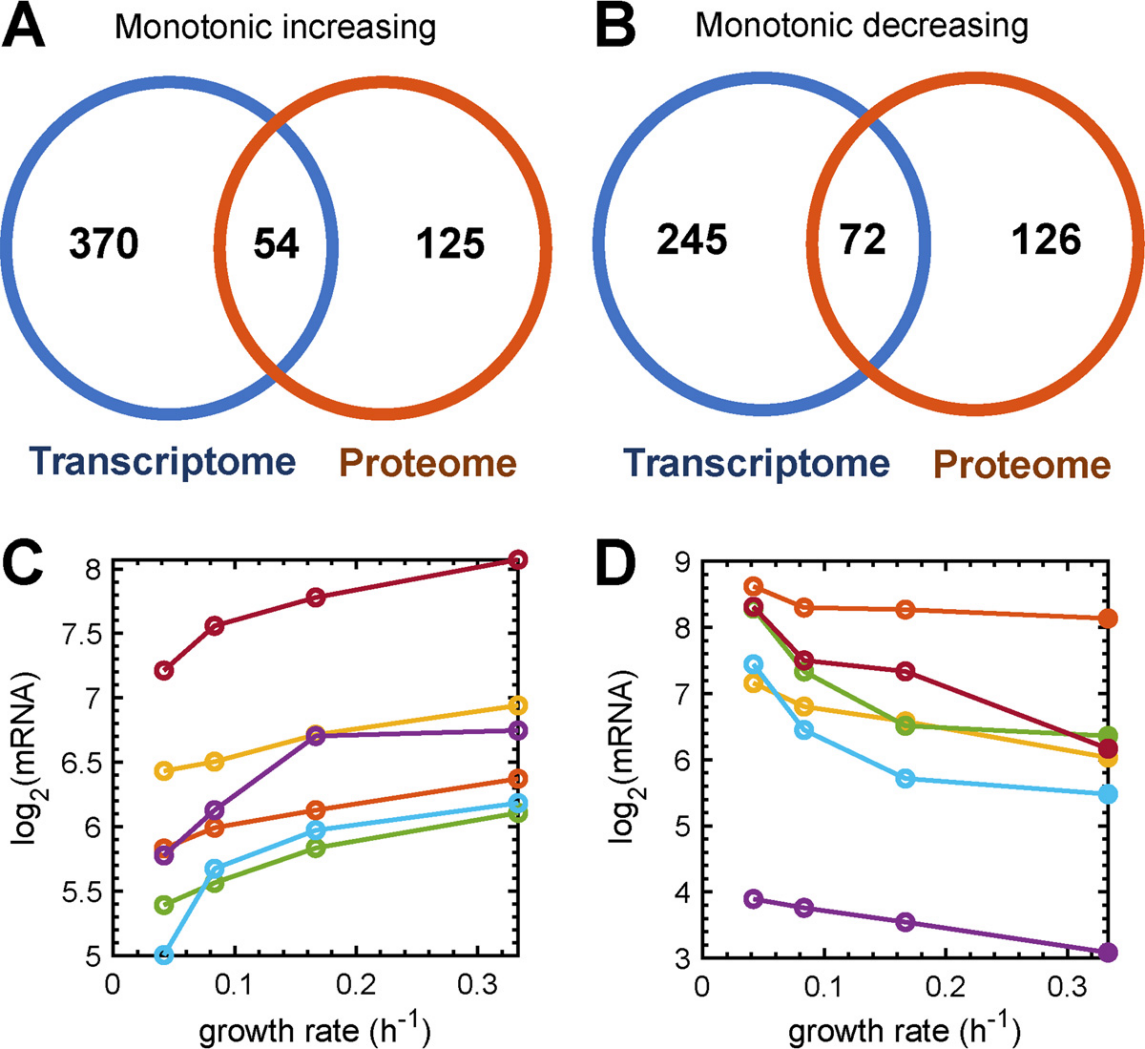
Identification of P. aeruginosa mRNAs and proteins that change in abundance monotonically with the growth rate. (A and B) Venn diagrams showing the overlap of monotonic increasing (A) and decreasing (B) levels as a function of the growth rate in both the transcriptome and proteome. (C) Representative genes that showed monotonic increasing gene expression as the growth rate increased. Genes listed from top to bottom are *PA14_61720* (red), *uraA* (yellow), *murI* (purple), *upp* (orange), *moeB* (green), and *prmC* (blue). (D) Representative genes that showed monotonic decreasing gene expression as the growth rate decreased. Genes listed from top to bottom are *PA14_66320* (orange), *gcvP1* (red), *arcD* (green), *arcC* (blue), *phaC2* (yellow), and *hutH* (purple).

The fact that we quantified gene expression at four growth rates provides a unique opportunity to assess gene expression across a range of growth rates. Thus, we next assessed the expression of the 2,362 genes differentially expressed at the highest and lowest growth rates (Data Set S1, sheet 3) across all growth rates, specifically focusing on whether these genes showed monotonic expression as the growth rate increased. Using statistical methods, we identified 424 genes whose expression increased and 317 genes whose expression decreased monotonically with increasing growth rates (Fig. 2A and C and Data Set S2, sheets 1 and 2). The majority of the genes that showed increased mRNA levels with increasing growth rates encode functions important for cell growth, including energy metabolism (F_o_F_1_ ATP synthase [*atpA*, *atpD*, *atpE*, *atpF*, *atpG*, and *atpH*]), oxidoreductase reactions (*nqrB*, *nqrC*, and *nqrF*), amino acid metabolism (arginine biosynthesis [*argB*, *argF*, *argG*, and *argH*]), metabolism of cofactors and vitamins (porphyrin metabolism [*cobB*, *cobC*, *cobI*, *cobJ*, *cobM*, *cobN*, *cobQ*, *cobT*, and *cobU*]), and fatty acid biosynthesis (*fabA*, *fabB*, *fabF1*, *fabG*, and *fabZ*). The expression of a large number of genes involved in translation also increased with increasing growth rates, including genes encoding 50S ribosomal proteins (*rplI*, *rplJ*, *rplK*, *rplL*, *rplW*, *rpmE*, and *rpmF*) and 30S ribosomal proteins (*rpsE*, *rpsF*, *rpsI*, *rpsL*, *rpsN*, *rpsQ*, and *rpsU*) and genes responsible for aminoacyl-tRNA biosynthesis (*alaS*, *glyQ*, *glyS*, *gatA*, *gatC*, *glnS*, *fmt*, *ileS*, *lysS*, *serS*, *thrS*, *trmD*, and *valS*). One hundred eighteen genes (37.2%) that showed a monotonic decrease in mRNA levels with increasing growth rates encode putative proteins of unknown function (Fig. 2C and Data Set S2, sheet 2). The remaining genes encode proteins involved in chemotaxis (*cheA*, *cheB*, and *cheW*), nitrate metabolism (*napA*, *napB*, *napC*, and *napD*), the *aa*_3_ cytochrome *c* oxidase (*coxA*, *coxB*, and *PA14_01310*), and type III secretion (*pcrG*, *pcrV*, *pscB*, *pscC*, *pscD*, *pscJ*, and *pscK*).

10.1128/mbio.03067-22.8 DATA SET S2 Assessment of mRNA and protein levels across growth rates. Download Data Set S2, XLS file, 0.4 MB. Copyright © 2022 Zhang et al. 2022 Zhang et al. https://creativecommons.org/licenses/by/4.0/ This content is distributed under the terms of the Creative Commons Attribution 4.0 International license.

We next asked if genes that showed monotonic mRNA expression across all growth rates also showed monotonic changes in protein levels (Data Set S2, sheets 9 to 12). Only 54 of the 424 genes whose mRNA levels increased monotonically with increasing growth rates also displayed monotonic increases in protein levels (Fig. 2B and Data Set S2, sheet 5). These genes encoded functions including small organic acid transport (*dctA*), amino acid metabolism (*trpB*, *lysC*, *lysA*, *hisC2*, and *purB*), lipid metabolism (*fabA*), nucleotide metabolism (*purB*), and ribosomal proteins (*rplI*, *rplJ*, *rplK*, *rpmE*, *rpsI*, *rpsL*, *rpsN*, and *rpsQ*). Only 72 of the 317 genes whose mRNA levels decreased with increasing growth rates also displayed monotonic decreases in protein abundances (Fig. 2B and Data Set S2, sheet 6), and 31 of these genes encode proteins of unknown function. Genes with known or putative functions included some involved in secretion (*pscC*, *pscJ*, *hcp3*, and *hsiJ3*), carbohydrate metabolism (*pslG*, *pslI*, *atoB*, *bkdA1*, *glgB*, and *prpD*), lipid metabolism (*glpK*, *pmtA*, and *fadE*), nitrate metabolism (*napC*), amino acid metabolism (*arcC* and *aspC*), translation (*fusA2*), and other functions (*algR*, *exsB*, *osmC*, and *osmE*). Our results reveal that while there are large numbers of genes whose mRNA levels change monotonically across four growth rates, only 13% of these genes also showed monotonic changes in protein levels.

We also developed statistical methods to discover genes whose expression followed more complex shapes. Eighty-five genes had high expression levels at both the highest and lowest growth rates but had low gene expression levels at the moderate growth rates, a pattern which we termed the “U-shape” pattern (Fig. S2A and Data Set S2, sheet 3). Many of these genes encode proteins involved in siderophore biosynthesis and pyochelin biosynthesis (*pchDCBA* and *pchEFG*) and were found to be organized into operons. Ninety-three proteins were shown to follow a U-shape pattern (Fig. S2C and Data Set S2, sheet 7), but only 4 of these genes overlapped those that showed U-shape mRNA levels. We also identified 242 genes that have low expression levels at both the highest and lowest growth rates but have high gene expression levels at moderate growth rates, termed the “upside-down U-shape” pattern (Fig. S2B and Data Set S2, sheet 4). No proteins showed an upside-down U-shape pattern (Fig. S2D and Data Set S2, sheet 8). These data indicate that while there are over 300 genes that show more complex U-shape expression patterns, U-shape protein patterns are very rare.

10.1128/mbio.03067-22.2 FIG S2 mRNAs and proteins with U-shape or upside-down U-shape expression patterns have little or no overlap. (A and B) Venn diagrams showing the overlap of U-shape (A) and upside-down U-shape (B) patterns of expression across growth rates in P. aeruginosa. (C and D) Representative genes that showed U-shape (C) and upside-down U-shape (D) patterns of gene expression. Download FIG S2, TIF file, 0.6 MB. Copyright © 2022 Zhang et al. 2022 Zhang et al. https://creativecommons.org/licenses/by/4.0/ This content is distributed under the terms of the Creative Commons Attribution 4.0 International license.

### mRNA and protein levels are positively correlated.

**FIG 3:**
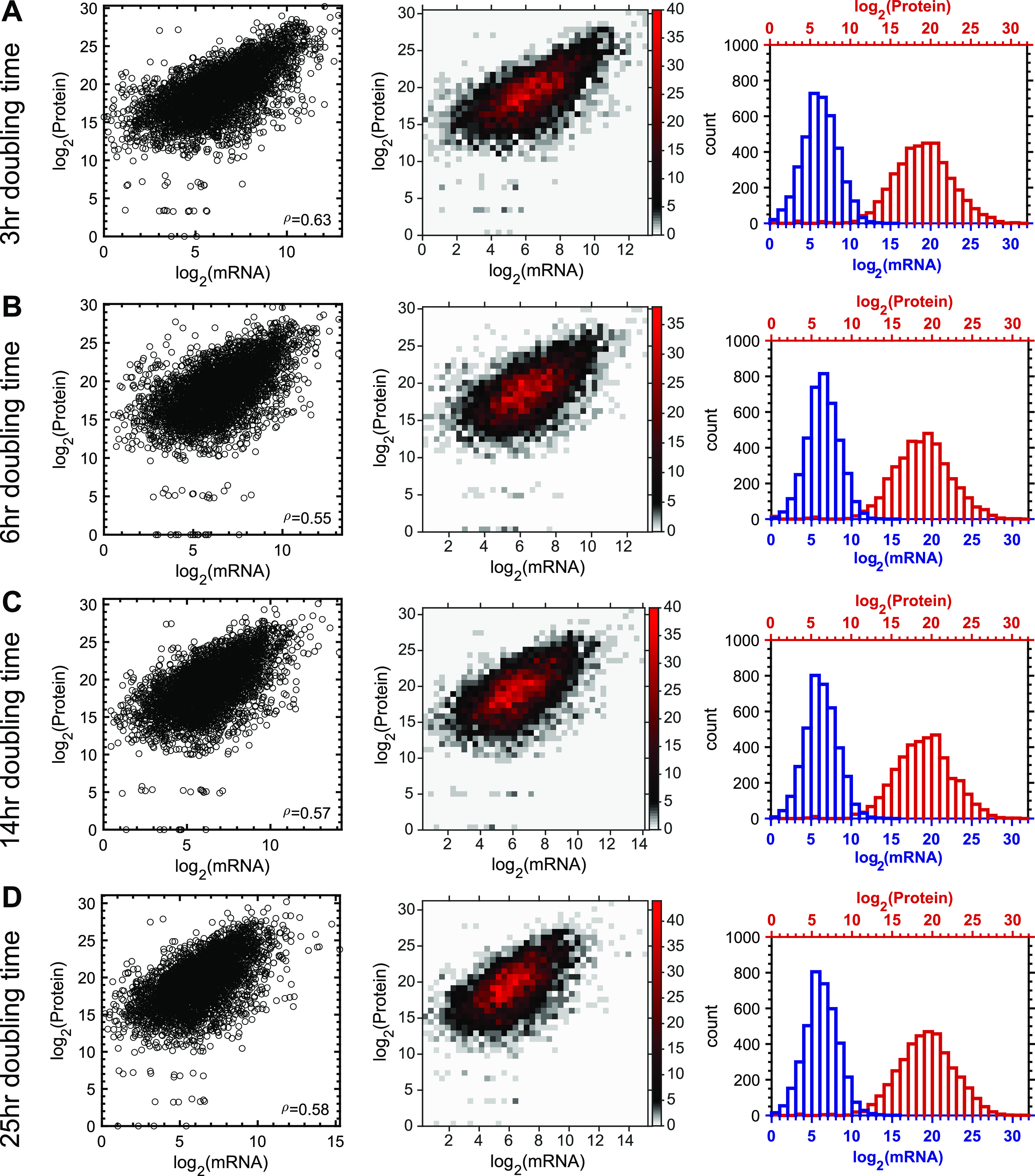
mRNA and protein are positively correlated at all growth rates. mRNA-protein relationships across a diverse range of growth rates spanning doubling times from 3 h to 25 h (A-D) are shown. Genes used for these analyses were detected at all growth rates in both the mRNA and protein expression data sets (*n* = 3,903). (Left) Scatterplots of mRNAs and proteins with associated Spearman rank correlation coefficients (ρ) demonstrating that mRNA and protein levels are positively correlated. (Middle) Binned scatterplots showing the relationship between measured protein abundances and mRNA abundances. The numbers of genes within each correlation are represented by the color scale in the right ordinate, where the number indicates the number of genes at a specific *x-y* coordinate. (Right) Distributions of mRNA and protein levels show that protein abundances vary to a greater extent than mRNA abundances.

While the relationship between mRNA and protein abundances has been studied in bacteria under single growth conditions (14–17, 20, 22, 23, 32), our data provide the opportunity to systematically evaluate if mRNA levels are a good proxy to predict protein abundances in P. aeruginosa across a range of steady-state growth conditions that cover *in situ* growth rates during chronic human infection. For these analyses, we restricted our analysis to 3,903 genes that were identified in both the mRNA and protein data sets at all growth rates. The paired data sets were restricted because the mass spectrometry (MS)-based proteomics method is a “nonamplification” process and is limited by the sensitivity and dynamic range of mass spectrometry; thus, it leads to the preferential detection of abundant proteins and peptides with robust ionization. For example, we found that membrane proteins (40) are less well detected in our proteomics data sets than predicted (*P* < 10^−41^ by Fisher’s exact test). Examination of the normalized distributions of detected mRNAs and proteins revealed a narrower distribution of mRNA than of protein, with measured protein abundances varying by ~10^6^-fold, while mRNA levels varied by ~10^3^-fold (Fig. 3A to D, right panels). Thus, when comparing the least and most abundant mRNAs, the magnitude of this difference is lower for mRNA than for protein. Whether this is a result of biological or methodological factors is not known, although this pattern is conserved across all growth rates.

We next quantified the correlation between protein and mRNA abundances by calculating the Spearman rank correlation coefficient (ρ) of the log_2_-transformed data, testing the hypothesis that gene expression levels correlate with protein levels (Fig. 3A to D, middle and right graphs). The correlations of mRNA and protein abundances were similar across growth rates (ρ = 0.55 to 0.63), indicating that the growth rate has little impact on this correlation. The coefficient of determination (*R*^2^) between mRNA and protein abundances revealed that, on average, 33% of the variability (28% to 39%) in protein levels can be explained by the variability in mRNA levels.

### Essential genes show higher positive correlations between mRNA and protein abundances.

**FIG 4:**
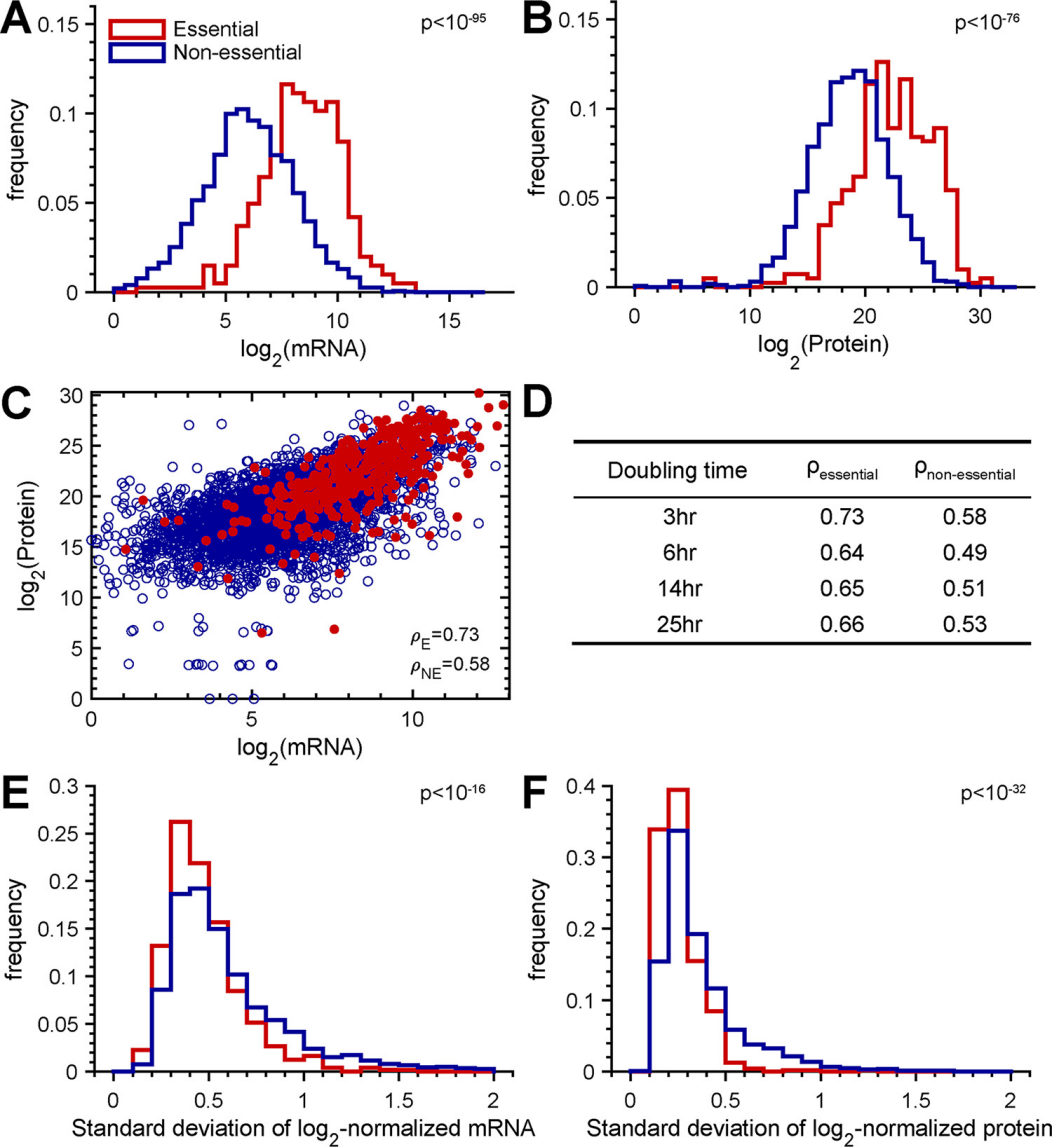
P. aeruginosa essential genes are highly expressed and have increased mRNA-protein correlations. (A and B) Distributions of mRNA (means ± standard deviations [SD], 8.4 ± 1.8 for essential genes and 6.0 ± 2.0 for nonessential genes) (A) and protein (means ± SD, 22.3 ± 3.5 for essential genes and 18.6 ± 3.4 for nonessential genes) (B) abundances for P. aeruginosa essential and nonessential genes at the 3-h doubling time reveal higher levels of both mRNA and protein for essential genes. (C) Scatterplot with associated Spearman rank correlation coefficient (ρ) for essential genes (red circles) (*n*_E_ = 484) and nonessential genes (blue circles) (*n*_NE_ = 3,419). (D) Spearman rank correlation coefficients for P. aeruginosa essential and nonessential genes at all growth rates. (E and F) Histograms of the standard deviations in mRNA (means ± SD, 0.48 ± 0.22 for essential genes and 0.62 ± 0.38 for nonessential genes) (E) and protein (means ± SD, 0.28 ± 0.32 for essential genes and 0.42 ± 0.49 for nonessential genes) (F) abundances for essential and nonessential genes demonstrate lower variance for essential genes than for nonessential genes. Histograms were constructed with a bin size of 0.05 using the variation in log_2_-normalized mRNA and protein levels from all growth rates. *P* values were calculated using a Wilcoxon rank sum test.

While we identified a genome-wide positive correlation between mRNA and protein abundances, we hypothesized that certain subsets of genes would exhibit stronger correlations. To test this hypothesis, we first assessed the RNA-protein relationships among genes and proteins differentially expressed at the highest and lowest growth rates and found similar correlations (Fig. S1). We then examined the correlation of mRNA and protein abundances for genes within TIGRFAM functional classifications. Protein synthesis showed a higher mRNA-protein correlation (ρ_ps_ = 0.79, *P* < 0.00001, and *N* = 150), while regulatory functions displayed a lower correlation (ρ_rf_ = 0.38, *P* < 0.0009, and *N* = 76). However, these analyses were largely limited by the number of genes present in each category, which spanned from tens to hundreds of genes. We next examined genes that are known to be essential for P. aeruginosa viability, defined using a P. aeruginosa PA14 transposon insertion mutant library (38, 41). Of the 434 essential genes, we used 404 that were present in both our mRNA and protein data sets. Both the mRNA (Fig. 4A) and protein (Fig. 4B) levels of the essential genes were significantly higher than those of the nonessential genes. In addition, the correlation between mRNA and protein levels of essential genes was significantly higher (0.64 to 0.73) than that of nonessential genes (0.49 to 0.58) across all growth rates (Fig. 4C and D) (*P* = 0.029 by a Wilcoxon rank sum test). We also demonstrated that the higher correlation of essential genes than of nonessential genes is not due to differences in gene numbers (Fig. S3) or expression levels (Fig. S4). In addition, despite the increased levels of both mRNA and protein, the essential genes displayed significantly lower variance than nonessential genes (Fig. 4E and F). These results demonstrate that genes essential for P. aeruginosa PA14 viability are highly expressed, are more stable in their expression levels, and have stronger mRNA-protein correlations across a range of growth rates.

10.1128/mbio.03067-22.3 FIG S3 Higher correlations between mRNA and protein abundances for essential genes than for nonessential genes are not due to differences in the numbers of genes. Shown are scatterplots of mRNA and protein abundances of P. aeruginosa grown at the 3-h doubling time for essential genes (A) and nonessential genes (B), which have been limited to the same number of genes by random subsampling (*N*_E_ = *N*_NE_ = 404). The Spearman rank correlation coefficient for essential genes (0.73) is still higher than the average correlation for nonessential genes subsampled 100 times (mean ± SD, 0.57 ± 0.039). Download FIG S3, TIF file, 0.4 MB. Copyright © 2022 Zhang et al. 2022 Zhang et al. https://creativecommons.org/licenses/by/4.0/ This content is distributed under the terms of the Creative Commons Attribution 4.0 International license.

10.1128/mbio.03067-22.4 FIG S4 Higher correlations between mRNA and protein abundances for essential genes than for nonessential genes are not due to comparable expression levels. Shown are scatterplots of mRNA and protein abundances in P. aeruginosa strains grown at the 3-h doubling time with their Spearman rank correlation coefficients for essential genes (A) and nonessential genes (B), which have been limited to include only genes with comparable mRNA and protein expression levels. Download FIG S4, TIF file, 0.5 MB. Copyright © 2022 Zhang et al. 2022 Zhang et al. https://creativecommons.org/licenses/by/4.0/ This content is distributed under the terms of the Creative Commons Attribution 4.0 International license.

### Identification of genes that have extreme protein-to-mRNA ratios.

**FIG 5:**
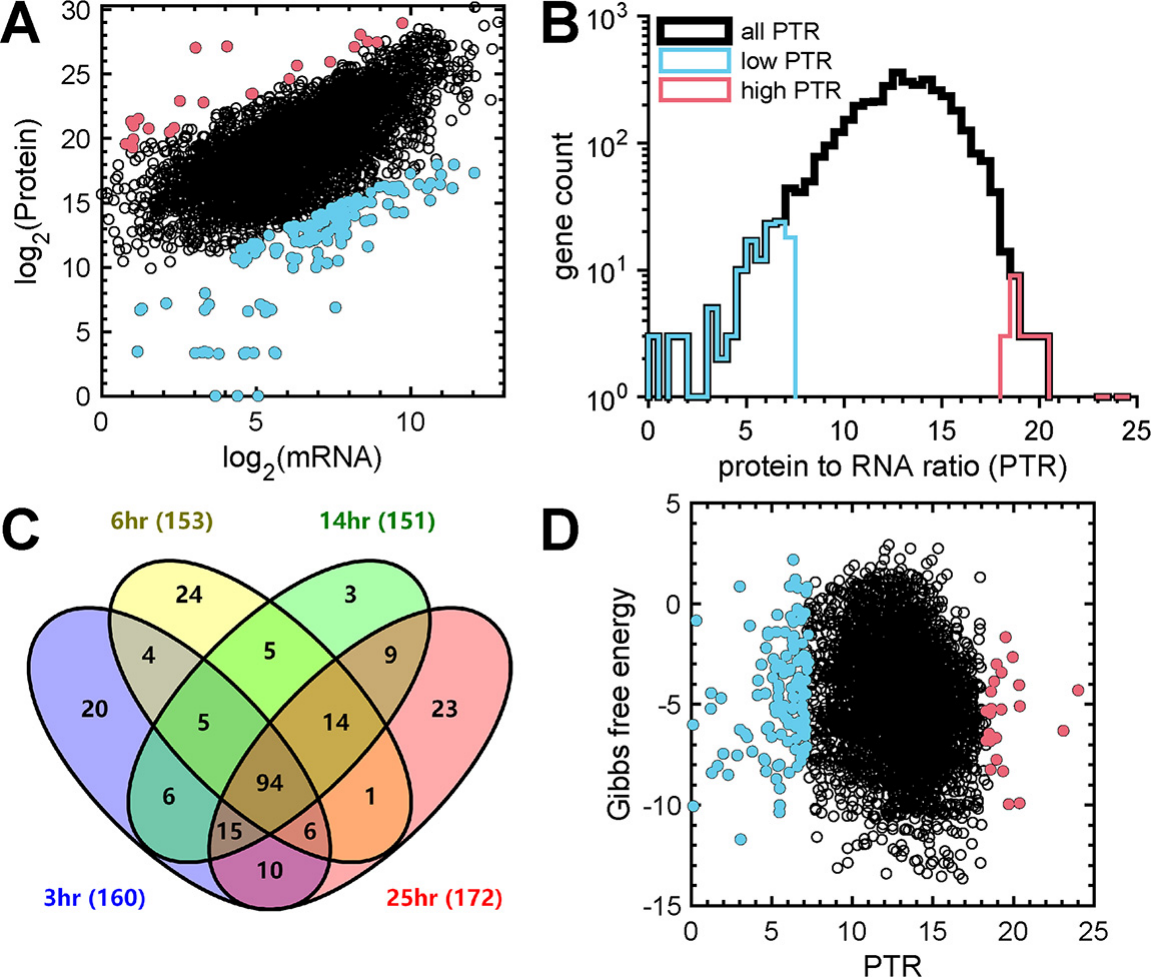
Identification of genes with poor mRNA-protein correlations. (A) Scatterplot of mRNA and protein abundances noting 160 genes with high (pink) or low (blue) PTRs at the 3-h doubling time. Genes with high and low PTRs were defined as being 2 standard residuals from the predicted correlation. (B) Distribution of genes with high (pink) and low (blue) PTRs at the 3-h doubling time. (C) Venn diagram of genes with poor mRNA-protein correlations across all growth rates. Ninety-four genes are shared at all growth rates. (D) Graph showing the predicted Gibbs free energy of the Shine-Dalgarno sequence binding to the 16S rRNA of the ribosome as a function of the PTR. The predicted binding energy of the Shine-Dalgarno sequence was weakly correlated with the PTR for all genes, including the 160 genes with high (pink) and low (blue) PTRs.

Which genes are most responsible for reducing the genome-wide correlation between mRNA and protein? To answer this question, we developed a statistical method to identify genes whose mRNA abundances were least predictive of protein levels. We identified genes (Fig. 5A, pink and blue dots) that most deviated from the best-fit linear regression lines of the genome-wide mRNA and protein levels at each growth rate (Fig. 3) using a statistical measure called the standardized residual. The standardized residual is a quantity that describes the difference among the observed protein levels minus the predicted protein levels where the prediction is based on the genome-wide linear regression among mRNAs and proteins (42). These low-predictive genes are categorized into two groups based on whether the differences in the protein-to-mRNA ratio (PTR) are higher or lower than expected (Fig. 5B and Data Set S3, sheets 1 to 4). The distribution of PTRs was positively skewed, with a long left tail containing 137 of the 160 genes (86%) with low PTRs at the 3-h growth rate (Fig. 5B and Data Set S3, sheet 4), indicating that it is more common to have genes with high mRNA levels but low protein levels than to have genes with low mRNA and high protein levels. There were 94 genes with extreme PTRs that were shared for all four growth rates and 40 genes that were shared for three of the four growth rates (Fig. 5C and Data Set S3, sheet 5), including genes encoding proteins involved in genetic information processing (*rodA*, *dcd2*, *rpoH*, *dnaE2*, and *bolA*), metabolism (*aroQ1*, *pabB*, *ubiA*, *aprI*, *phzE1*, and *trxB2*), and environmental information processing (*fha1*, *secG*, *tssF1*, and *nppC*) (33). There were 70 genes that were specific for a single growth rate (Data Set S3, sheets 6 to 9) and another 35 genes that were shared at only two growth rates, including genes involved in siderophore biosynthesis (*pvdL*, *pvdH*, *PA14_33550*, *PA14_33560*, *PA14_33610*, *fpvA*, *pvdE*, *pvdO*, *pvdN*, and *pvdA*). Enrichment analysis did not reveal any significant enrichment of TIGRFAM functional categories among genes that are shared or not shared between growth rates (*P* < 0.05 by Fisher’s exact test), likely due to the fact that most genes with extreme PTRs encode proteins of unknown function.

10.1128/mbio.03067-22.9 DATA SET S3 Identification of genes whose mRNA abundances were least predictive of protein levels at each growth rate. Download Data Set S3, XLS file, 0.09 MB. Copyright © 2022 Zhang et al. 2022 Zhang et al. https://creativecommons.org/licenses/by/4.0/ This content is distributed under the terms of the Creative Commons Attribution 4.0 International license.

We next explored the potential mechanisms that lead to genes with extreme PTRs. We first tested if genes with low PTRs are enriched for secreted proteins, with the rationale being that our proteomic experiments did not quantify secreted proteins. We did not find an enrichment of extracellular proteins (*P* = 0.71 to 1.0 by Fisher’s exact test) in genes with low PTRs across all growth rates. We next tested for the enrichment of proteins that are not well detected by mass spectrometry, but again, we did not observe an enrichment of small, <10-kDa proteins (*P* = 1.0 by Fisher’s exact test) or membrane proteins (*P* = 1.0 by Fisher’s exact test) in genes with low PTRs. We then assessed whether genes with extreme PTRs could be explained by the sequence of the ribosome binding site (also referred to as the Shine-Dalgarno sequence), which has been shown to be important for the efficiency of translation initiation in Escherichia coli (43). For this analysis, we calculated the predicted Gibbs free energy of binding between the Shine-Dalgarno sequence of each gene and the 16S rRNA component of the ribosome, with the hypothesis that genes with a high PTR would have strong binding (low Gibbs free energy) and genes with a low PTR would have weak binding (high Gibbs free energy) (44). However, the Gibbs free energy showed weak correlations with protein levels (ρ = −0.12 to −0.09) and PTRs (ρ = −0.18 to −0.17) (Fig. 5D), explaining only ~1.0% (0.80% to 1.4%) of the variability in PTR levels and ~2.1% (2.0% to 2.3%) of the variability in protein levels across all growth rates. These data indicate that the secreted proteins, proteins poorly detected by mass spectrometry, and the Shine-Dalgarno sequence are not primary drivers of the PTRs of these extreme genes.

### Using a gene-specific RNA-to-protein conversion factor to increase the predictability of protein from mRNA levels.

In addition to assessing the correlation between protein and mRNA levels for each gene, a goal of this work is to determine if we can improve the predictability of some poorly correlated genes using an RNA-to-protein (RTP) conversion factor. The basic idea is to identify poorly correlated genes that have consistent PTRs across growth conditions by calculating an inconsistency factor and then devising a conversion factor that can be applied to mRNA measurements to improve predictions of protein levels (18). The RTP conversion factor is a gene-specific conserved quantitative metric and is calculated by assessing the protein-to-mRNA ratios across all growth conditions for those genes identified using consistency factors. In addition to the chemostat transcriptomic and proteomic data generated in this study, we also used paired, publicly available transcriptomic and proteomic data for P. aeruginosa strains PA14 and PAO1 during batch culture growth in a medium (SCFM [synthetic CF sputum medium]) (32) that mimics the nutritional environment of the CF lung (38, 45). The rationale for including these data is that they were acquired under medium conditions and at a growth rate (40-min generation time) different from those used in the chemostat experiments. They also included data from a different P. aeruginosa strain (PAO1). These additional data provide the opportunity to assess the robustness of RTP conversion factors.

We identified 309 poorly correlated genes that had consistent RNA levels, protein levels, and PTRs (Fig. S6) and calculated an RTP conversion factor for each of these genes (Data Set S4). Examination of the functions of these genes revealed that many encode proteins important for central carbon metabolism (*rpiA*, *tktA*, *pgk*, *eno*, *tal*, *aceA*, *idh*, *sucB*, *acnA*, *aceF*, *mqoA*, and *mqoB*), purine biosynthesis (*ndk*, *guaA*, *purT*, *adk*, *purF*, *purB*, *purC*, *purM*, *purD*, *purA*, and *purK*), branched-chain amino acid biosynthesis (*ilvD*, *leuA*, *leuB*, *ilvH*, *ilvI*, and *ilvE*), and aminoacyl-tRNA biosynthesis (*glyS*, *fmt*, *leuS*, *hisS*, *lysS*, *metG*, *gltX*, *thrS*, *serS*, *cysS*, *aspS*, *alaS*, and *argS*). A total of 114 out of 309 genes (~40%) are essential in P. aeruginosa, indicating that many poorly correlated essential genes have consistent PTRs that allow the calculation of a robust RTP conversion factor.

10.1128/mbio.03067-22.6 FIG S6 Application of inconsistency factors to threshold genes appropriate for RTP conversion factors. Shown are cumulative distribution function curves (CDF) for inconsistency factors applied to chemostat transcriptomes (green), chemostat proteomes (blue), and PTRs under all conditions (red). Download FIG S6, TIF file, 0.2 MB. Copyright © 2022 Zhang et al. 2022 Zhang et al. https://creativecommons.org/licenses/by/4.0/ This content is distributed under the terms of the Creative Commons Attribution 4.0 International license.

10.1128/mbio.03067-22.10 DATA SET S4 Increased predictability of protein levels from mRNA levels using gene-specific RTP conversion factors and table of raw abundance values from proteomics experiments. Download Data Set S4, XLS file, 1.1 MB. Copyright © 2022 Zhang et al. 2022 Zhang et al. https://creativecommons.org/licenses/by/4.0/ This content is distributed under the terms of the Creative Commons Attribution 4.0 International license.

**FIG 6:**
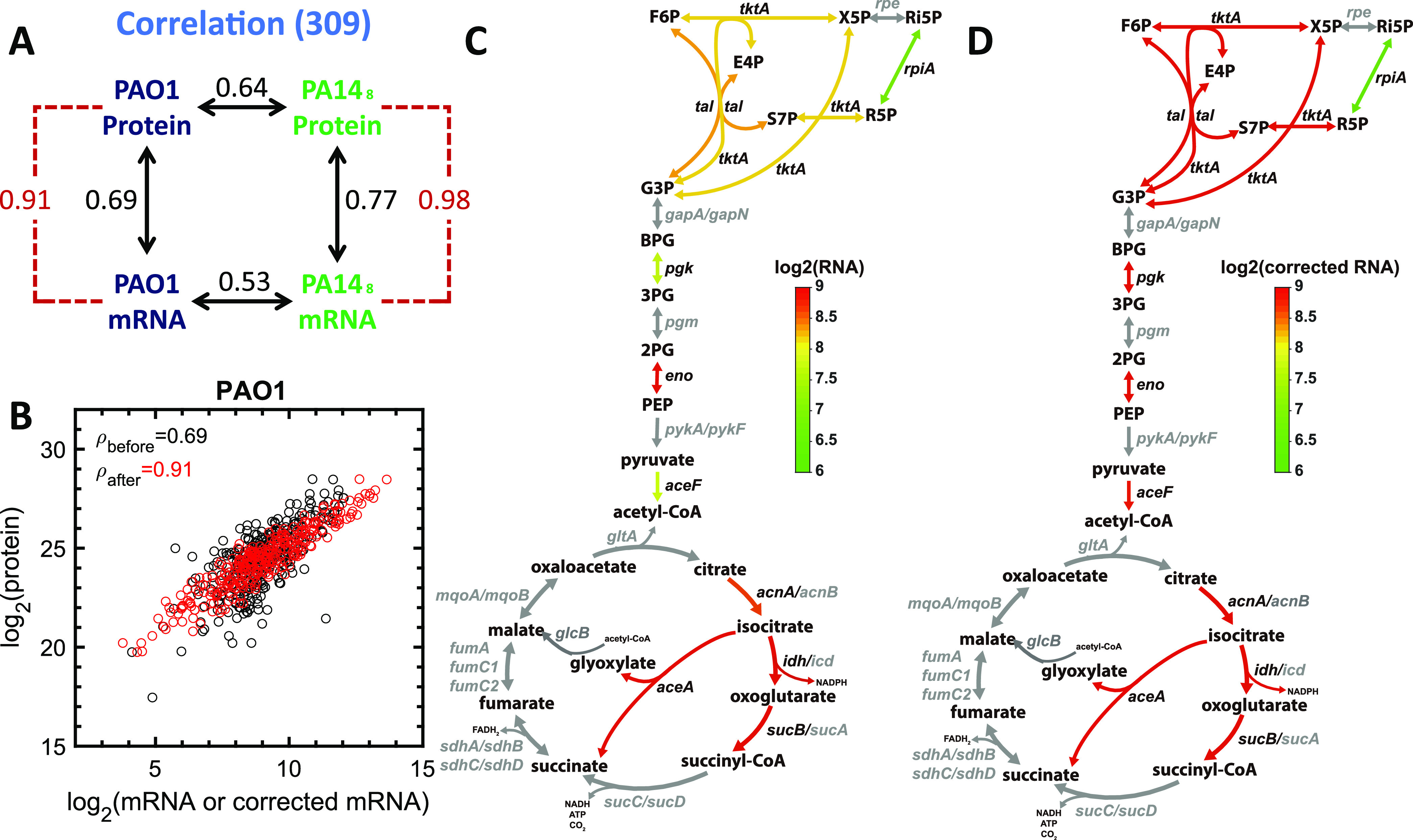
Application of gene-specific RTP conversion factors to P. aeruginosa transcriptomes from other strains, growth conditions, and human CF infections. (A) Prediction of protein levels by the application of the RTP conversion factors to mRNA levels of P. aeruginosa strain PAO1 grown in a synthetic CF sputum medium (SCFM) and strain PA14 grown in chemostats. The application of the RTP conversion factors improved the mRNA-protein Spearman rank correlation coefficients for PAO1 from 0.69 to 0.91 and for PA14 from 0.77 to 0.98. (B) Scatterplot of RNA-protein relationships among measured mRNA and protein levels (black circles) and mRNA corrected by the application of the RTP conversion factors and measured protein levels (red circles). Data are from P. aeruginosa strain PAO1 grown in a synthetic CF sputum medium. The associated Spearman rank correlation coefficients (ρ) among RNA and protein abundances increased from 0.69 to 0.91 after the application of the RTP conversion factors to mRNA levels. (C and D) Central metabolic pathway, with colored arrows showing predicted metabolic fluxes using mRNA levels from P. aeruginosa human CF infection transcriptomes (C) and mRNA levels from P. aeruginosa human CF infection transcriptomes after applying the RTP conversion factor (D). Gray indicates the absence of genes or proteins detected in the data sets. F6P, fructose-6-phosphate; X5P, xylulose-5-phosphate; Ri5P, ribulose-5-phosphate; E4P, erythrose-4-phosphate; S7P, sedoheptulose-7-phosphate; R5P, ribose-5-phosphate; G3P, glyceraldehyde 3-phosphate; BPG, 1,3-bisphosphoglycerate; 3PG, 3-phosphoglycerate; 2PG, 2-phosphoglycerate; ATP, adenosine triphosphate; NADH, nicotinamide adenine dinucleotide; NADPH, nicotinamide adenine dinucleotide phosphate; PEP, phosphoenolpyruvate; FADH_2_, reduced flavin adenine dinucleotide.

We next assessed the utility of the RTP conversion factor. As expected, the application of the RTP to both the P. aeruginosa PAO1 and PA14 mRNA data sets improved the mRNA predictivity of protein levels (Fig. 6). Applying the RTP conversion factors to the mRNA abundances of batch-grown P. aeruginosa PAO1 in SCFM and strain PA14 grown in chemostats improved the mRNA-protein Spearman rank correlation coefficients for PAO1 from 0.69 to 0.91 and for PA14 from 0.77 to 0.98 (Fig. 6A and B). To test the robustness of this improvement, we performed leave-one-out model validation of our RTP conversion factors. In this analysis, we left one of our six test data sets out of the model and then applied these estimated conversion factors to the left-out transcriptome to determine if the corrected transcriptome was more predictive of measured protein levels (Table 1). We discovered that the correlations among the predicted RTP-corrected mRNA and protein levels are significantly higher than the observed mRNA-to-protein correlations (*P* < 0.003 by a Wilcoxon rank sum test) under all conditions. The prediction of the left-out chemostat data was slightly better than that of the batch culture SCFM data (Table 1), likely because our data set is dominated by chemostat data. However, the mRNA predictabilities of protein levels of the SCFM batch data for both strains PA14 and PAO1 were also highly improved (Table 1).

**TABLE 1:** Leave-one-out model validation of RTP conversion factors

Strain^*a*^	Growth conditions^*b*^	*N*_gene_ ^*c*^	ρ_measured_^*d*^	ρ_corrected_^*d*^
PA14	Chemostat, MOPS-succinate, 25 h	289	0.76	0.95
PA14	Chemostat, MOPS-succinate, 14 h	280	0.73	0.97
PA14	Chemostat, MOPS-succinate, 6 h	298	0.74	0.97
PA14	Chemostat, MOPS-succinate, 3 h	270	0.77	0.97
PAO1	Shaking flask, SCFM	381	0.72	0.89
PA14	Shaking flask, SCFM	388	0.72	0.90

a Strain of P. aeruginosa.

b Growth conditions that were left out of RTP conversion factor calculation and then corrected using the RTP conversion factor.

c *N*_gene_, number of genes chosen for individual leave-one-out models.

d Spearman rank correlation coefficient of measured (ρ_measured_) and RTP-corrected (ρ_corrected_) mRNA-protein levels.

### Application of RTP conversion factors to P. aeruginosa human infection transcriptomes.

Since methods for assessing bacterial physiology during human infection are limited, the ability to perform RNA-seq on P. aeruginosa during human infection is highly informative. A prime motivator of this work was to develop RTP conversion factors to enhance the physiological relevance of these transcriptomes, with the goal of enhancing our understanding of the functions of P. aeruginosa during human infection. Thus, we applied the RTP conversion factors to transcriptomes gathered from P. aeruginosa infecting the human CF lung (4, 5). The results revealed that central metabolic pathways were significantly affected by the application of the RTP conversion factor, including an increase in enzymes of the pentose phosphate pathway (Fig. 6C and D). However, other pathways, including tricarboxylic acid enzymes, were minorly impacted by the application of the RTP conversion factor (Fig. 6C and D and Fig. S5).

10.1128/mbio.03067-22.5 FIG S5 RTP conversion factors can improve predicted metabolic fluxes from human infection transcriptome data. Important metabolic pathways are shown, with colors showing predicted metabolic fluxes using mRNA levels from P. aeruginosa human CF infection transcriptomes (top or left) and corrected mRNA levels (bottom or right) after applying the RTP conversion factor. Gray indicates the absence of genes or proteins detected in the data sets. These pathways include important genes for bacterial physiology, such as aminoacyl-tRNA biosynthesis (A), branched-chain amino acid biosynthesis (B), and purine metabolism (C). Download FIG S5, PDF file, 0.4 MB. Copyright © 2022 Zhang et al. 2022 Zhang et al. https://creativecommons.org/licenses/by/4.0/ This content is distributed under the terms of the Creative Commons Attribution 4.0 International license.

## DISCUSSION

The goal of this work was to explore the impact of the growth rate on mRNA and protein contents in P. aeruginosa. Our highly controlled chemostat experiments allowed us to comprehensively quantify the mRNA and protein compositions of cells at steady state across a range of growth rates observed during human infection. It is clear that the growth rate significantly alters the protein and mRNA compositions of P. aeruginosa, with hundreds of mRNAs and proteins being differentially present in cells growing at high and low growth rates (Fig. 1). Both mRNAs and proteins from genes encoding ribosomal proteins were elevated in fast-growing cells. This is an expected result because ribosome numbers generally increase in faster-growing bacteria (46–48). Some of these ribosomal proteins were members of a group of 54 genes in which both mRNA and protein levels increased monotonically with the growth rate (see Data Sets S1 and S2 in the supplemental material). These genes, along with 72 genes for which mRNA and protein levels decreased monotonically with the growth rate (Fig. 2 and Data Sets S1 and S2), have the potential to be used to predict the average growth rate of P. aeruginosa populations in complex bacterial communities in which the growth rate cannot easily be measured. One aspect of our future work is to leverage these genes to predict growth rates in P. aeruginosa human infections using linear regression and deep-learning approaches.

Like many other studies in organisms from all domains of life (13–27), we found that mRNA and protein levels are generally positively correlated. Correlations were similar across growth rates, with the highest Spearman rank correlation coefficient (ρ = 0.63) being observed at the highest growth rate (Fig. 3). However, there are a number of genes that showed poor correlations (Fig. 3). While the majority of the most poorly correlated genes have low PTRs (Fig. 5B and Data Set S3, Sheet 1-4), these genes were not enriched for secreted proteins, indicating that the low-PTR genes cannot be explained by the fact that our proteomics experiments did not quantify secreted proteins. We also assessed protein properties that might reduce their detection by mass spectrometry (49–52) and found that low-PTR genes are not enriched for membrane or small (<10-kDa) proteins, nor could the extremely low- or high-PTR genes be fully explained by the Shine-Dalgarno sequence (Fig. 5D), which is consistent with the results of previous studies in which the Shine-Dalgarno sequence had little explanatory power for genes with extreme PTRs (32, 33). Thus, while the mechanism is unknown, it is likely that many of these genes are the targets of posttranscriptional regulation either via mRNA/protein degradation or by small regulatory RNAs.

The observation that the abundances of mRNAs from P. aeruginosa essential genes were more predictive of protein levels than nonessential genes (Fig. 4) provides new insights into these conserved genes. Consistent with the results of previous studies in yeast and human cell lines (53, 54), we also noted that essential genes are more highly expressed than nonessential genes. mRNAs from only 14 of the 404 essential genes were present at levels below 2^5^, and only 10 of 404 essential proteins were detected at levels below 2^15^ (Fig. 4C). One potential explanation for the high protein predictability of essential gene mRNA is that the high levels of mRNA and protein reduce the detection bias. However, nonessential genes with protein and mRNA levels similar to those of essential genes have lower correlations (Fig. S4). Instead, we propose that essential genes have stable PTRs as they serve core functions in the cell and act as hubs in protein-protein interaction networks, similar to those observed in human cells (53). As such, we predict that most essential genes are not subject to significant posttranscriptional regulation but instead rely on mRNA levels to modulate protein numbers.

Previous work has shown that gene-specific RTP conversion factors can be used to better predict protein abundances from mRNA levels across human tissues and cell lines (18). Here, we identified 309 P. aeruginosa genes that have consistent RTP conversion factors across strains, media, and growth rates (Fig. 6B and Data Set S4). Notably, the application of these correction factors to RNA-seq data acquired from a different strain of P. aeruginosa grown under nonchemostat conditions improved the protein predictivity of mRNA levels (Table 1). We anticipate that this RTP conversion factor approach can be applied to many more than the 309 genes identified in this study. Here, our identification of these genes was limited by the poor depth of coverage of the proteomics data from the previous study (32), where only 703 proteins were detected. Overall, this approach appears to be robust, and further work generating high-coverage, simultaneous transcriptomes and proteomes from additional P. aeruginosa strains under a range of growth conditions will strengthen the identification of appropriate genes and the robustness of our RTP conversion factor approach.

This study furthers our recent work using RNA-seq to assess the physiology of bacteria during human infection (4, 5, 7–9, 55). While there are challenges in using mRNA levels to predict protein levels in microbial communities due to the poor mRNA-protein correlations of some genes, this work provides a corrective framework to increase the predictability of mRNA by assessing protein-RNA relationships across strains and growth conditions. Our discovery that essential genes have stronger mRNA-protein correlations is encouraging given that many of these genes are involved in fundamental processes critical for pathogenesis. This allows the protein levels of these genes to be inferred from transcriptomic data with high accuracy in relevant environments (i.e., human infection), which we suggest will be valuable for efforts focused on the development of antimicrobials targeting these essential functions.

In sum, we propose that there are significant gaps in knowledge regarding the physiology and behavior of microbes during human infection that hamper progress in diagnosing and treating infectious diseases. For example, a better understanding of the biology of infection could be used to improve the physiological relevance of the preclinical model systems needed to develop new therapeutics. Transcriptomic studies, which are some of the few methods that can be performed on complex microbial communities *in situ*, are leading the way in generating this knowledge. It is therefore critical to understand and address the limitations of these data sets, as we have done here. While it is likely that technological advancements will allow robust, high-throughput assessments of the microbial proteome during human infection, these approaches are likely years away from generating sufficient data for robust analyses. Thus, our approach fills an important gap in knowledge for understanding bacterial physiology in native environments.

## MATERIALS AND METHODS

### Strains, media, and growth conditions.

Pseudomonas aeruginosa strain UCBPP-PA14 (PA14) (56, 57) was routinely grown on lysogeny broth-Miller (LB) agar plates and LB broth (Sigma-Aldrich) at 37°C. The chemostat growth medium was a morpholinepropanesulfonic acid (MOPS)-buffered minimal medium (58) with 10 mM succinate as the sole carbon source (MOPS-succinate). The chemostat growth chamber was composed of a 300 ml Berzelius beaker (Kimble) containing 100 ml of culture volume that was continuously stirred and aerated with filtered air as previously described (58). Chemostat cultures were inoculated, monitored, and grown to steady state in a warm room at 37°C. The flow rates were set to 3.9 mL/h, 7.3 mL/h, 16.6 mL/h, and 30.3 mL/h using a peristaltic pump, which corresponded to generation times of 25.3 h (referred to as 25 h), 13.8 h (referred to as 14 h), 6 h, and 3.3 h (referred to as 3 h). The chemostat was maintained at steady state for 48 h before sampling.

### Sample collection and library preparation for RNA-seq.

A 5-mL sample of the culture was taken from at least two chemostat experiments with 4 replicates in total and mixed with RNAlater, which was incubated overnight at 4°C and then stored at −80°C. RNA-seq libraries were prepared as previously described for *in vitro* samples (5). rRNA was depleted using the MICROBExpress bacterial mRNA enrichment kit (Sigma). The depleted RNA was fragmented for 2 min with the NEBNext magnesium RNA fragmentation module kit, and cDNA libraries were prepared using the NEBNext multiplex small RNA library prep kit (New England BioLabs). The final RNA concentration for each sample was assessed with a Qubit dsDNA HS (double-stranded DNA high-sensitivity) assay kit (Thermo) and the high-sensitivity Bioanalyzer kit (Agilent). Libraries were sequenced at the Molecular Evolution Core at the Georgia Institute of Technology on an Illumina NextSeq500 platform using 75-bp single-end runs.

### Bioinformatic analysis for RNA-seq.

RNA-seq reads were mapped and analyzed as previously described by the Whiteley group (5). Gene counts were tallied to the coding sequences (CDSs) using the R package featureCounts (available under the subread package) using the strand-specific option. For correlational analyses, raw data were normalized using the reads per million mapped reads (RPM) method. Differential expression between the two extreme growth rates (see Data Set S1 in the supplemental material) was determined using DESeq2 (Bioconductor). Transcripts were considered differentially increased or decreased in abundance only when their *P*_adj_ value was <0.05 and their |log_2_ fold change| >2. All figures were created in MATLAB using the scatter, histogram, imagesc, and PlotPub functions.

### Sample collection and preparation for proteomics mass spectrometry.

For each sample, 5 mL of the chemostat culture was collected on ice, followed immediately by centrifugation at 4°C at 14,000 × *g* for 5 min. The supernatant was removed, and cells were washed once in 1 mL of ice-cold sterile phosphate-buffered saline (PBS). Cells were pelleted again, the supernatants were removed, and the cell pellets were stored at −80°C. To allow relative comparisons of proteins between samples, proteins in all samples were labeled, pooled, and quantified in a single run by the Georgia Institute of Technology’s Systems Mass Spectrometry Core Facility. Proteins were extracted from frozen cell pellets using a urea lysis buffer and digested with trypsin overnight. Peptides were labeled with tandem mass tag (TMT) isobaric labels (Thermo Fisher Scientific), pooled, and separated by HPLC (high-pressure liquid chromatography; Beckman). Specifically, a total of 120 fractions were collected and pooled. Both the unfractionated and the pooled fractions were analyzed by nano-liquid chromatography-tandem mass spectrometry (nano-LC/MS-MS) using the Dionex nanoLC system coupled to a Q-Exactive Plus mass spectrometer (Thermo Scientific). Reverse-phase chromatography was performed using an in-house-packed column (40-cm long by 75-μm internal diameter by 360-μm outer diameter with ReproSil-Pur 120 C_18_-AQ 1.9-μm beads [Dr. Maisch GmbH]) and a 120-min gradient. Mass spectrometry was performed in a data-dependent mode, including a full MS1 scan and 12 MS2 scans. The MS1 scan was performed using parameters with a 70,000 resolution, 3 × 10^6^ automatic gain control (AGC), and a 20-ms maximal ion time. The MS2 scans were performed at a 35,000 resolution, 5 × 10^4^ AGC, a 120-ms maximal ion time, high-energy collisional dissociation (HCD), 30% normalized collision energy, a 1.6-*m/z* isolation window with 0.3-*m/z* offsets, and a 20-s dynamic exclusion.

### Protein search parameters, mass spectrometry quantification, and data analysis.

Raw mass spectrometry data files were processed with Mascot (v2.6; Matrix Science) and Proteome Discoverer (v2.1; Thermo Fisher Scientific). Data were searched against the predicted P. aeruginosa PA14 proteome (www.pseudomonas.com), in addition to using the common repository of adventitious proteins (cRAP) v1.0. The data were searched with MS peptide tolerances of 10 ppm and an MS/MS tolerance for identification of 100 millimass units (mmu) for the precursor. Quantification was achieved by calculating the sum of the centroided reporter ions within a ±2-mmu window around the expected *m/z* for each of the 4 TMT reporter ions. The results were then exported to Microsoft Excel for further data interpretation and statistical analysis. Proteins that were not detected in all channels were excluded and then log_2_ transformed. Differential protein expression was statistically determined using DESeq2 (Bioconductor). Proteins were considered differentially increased or decreased in abundance only when their log_2_ fold change was greater than 2 or less than −2, respectively, and their adjusted *P* value was <0.05. Venn diagrams were created with Venny (59).

### Shape analysis: monotonic genes.

To identify mRNA or proteins whose levels changed monotonically with the growth rate, we evaluated the mRNA and protein dynamics across all four growth rates using two steps. First, for both mRNA and protein, we selected genes that showed consistent positive or negative changes in levels using pairwise comparisons of the four generation times (3 h-6 h, 6 h-14 h, and 14 h-25 h). Second, we performed differential gene expression analysis between the highest and lowest growth rates to ensure that the trend of increasing/decreasing levels is significant under these two extreme conditions using a *P*_adj_ value of ≤0.05.

### Shape analysis: U-shape and upside-down U-shape patterns.

To identify mRNA and proteins with U-shape and upside-down U-shape patterns, for each gene, we performed four two-sample one-tailed *t* tests comparing mRNA levels at four pairs of growth rates: 3 h-6 h, 3 h-14 h, 6 h-25 h, and 14 h-25 h. Genes were defined as having U-shape patterns if the following conditions were met for mRNA levels (*P* ≤ 0.05): 3 h > 6 h, 3 h > 14 h, 6 h < 25 h, and 14 h < 25 h (Data Set S3). Genes were selected as having upside-down U-shape patterns if the following conditions were met for mRNA levels (*P* ≤ 0.05): 3 h < 6 h, 3 h < 14 h, 6 h > 25 h, and 14 h > 25 h (Data Set S3).

### RTP conversion factor calculation and application to human P. aeruginosa transcriptomes.

In addition to our chemostat data, transcriptome and proteome data from 703 genes identified previously by Kwon et al. (32) were also used for RTP conversion factor calculation. All data were normalized to identical depths for analysis.

To identify conserved metrics to predict protein levels from mRNA levels, we calculated an inconsistency factor as previously described (32) to identify genes with consistent RNA levels, protein levels, and PTRs. The inconsistency factor was calculated as the sum of the positive distances between data points of individual data sets and the mean value divided by the number of conditions:

$$\text{inconsistency factor}=\frac{\sum^{\;}|x_{i}-\bar{x|}}{n}$$

We identified 309 genes that showed consistent RNA levels, protein levels, and PTRs using an inconsistency factor cutoff of 0.6 across all experimental conditions (Fig. S6). For these genes, the RTP conversion factor was calculated as the average PTR across all conditions.

To assess the robustness of the RTP conversion factors, we performed two analyses. First, we calculated the predicted protein level for each of the 309 genes by multiplying the mRNA level of each gene by its RTP conversion factor. Next, we calculated the Spearman rank correlation coefficient for the predicted protein levels compared to the observed protein levels. Second, we performed a leave-one-out analysis for all six experimental conditions. In each case, five experimental conditions were used for the calculation of RTP conversion factors. These were then applied to the data from the left-out experimental condition by multiplying the mRNA level of each gene by the gene-specific conversion factor. The predicted protein levels were then compared to the measured protein levels for the left-out experimental condition using a Wilcoxon rank sum test.

RTP conversion factors were applied to P. aeruginosa mRNA levels quantified previously from human-derived samples from cystic fibrosis lung infections (4, 5). The corrected mRNAs are calculated based on the predicted protein levels and the best-fit regression line that linearly associated genome-wide mRNA and protein levels. Only 308 of the 309 RTP conversion factors were used for this analysis, as the mRNA for one gene was not detected in the cystic fibrosis lung infection transcriptomes. Pathways predicted from the P. aeruginosa CF transcriptomes and corrected with the RTP conversion factor were constructed as previously described (7, 38, 60).

### Data availability.

Raw RNA-seq data are available in the NCBI Sequence Read Archive under BioProject accession number PRJNA867727, and raw proteomics data have been deposited to the Mass Spectrometry Interactive Virtual Environment (MassIVE) database with the identifier MSV000090126.
