# Supplementary figures and images for: Impact of Growth Rate on the Protein-mRNA Ratio in Pseudomonas aeruginosa
Source: mBio. 2022 Dec 8;14(1):e03067-22. doi: 10.1128/mbio.03067-22 (PMC9973009; doi:10.1128/mbio.03067-22)

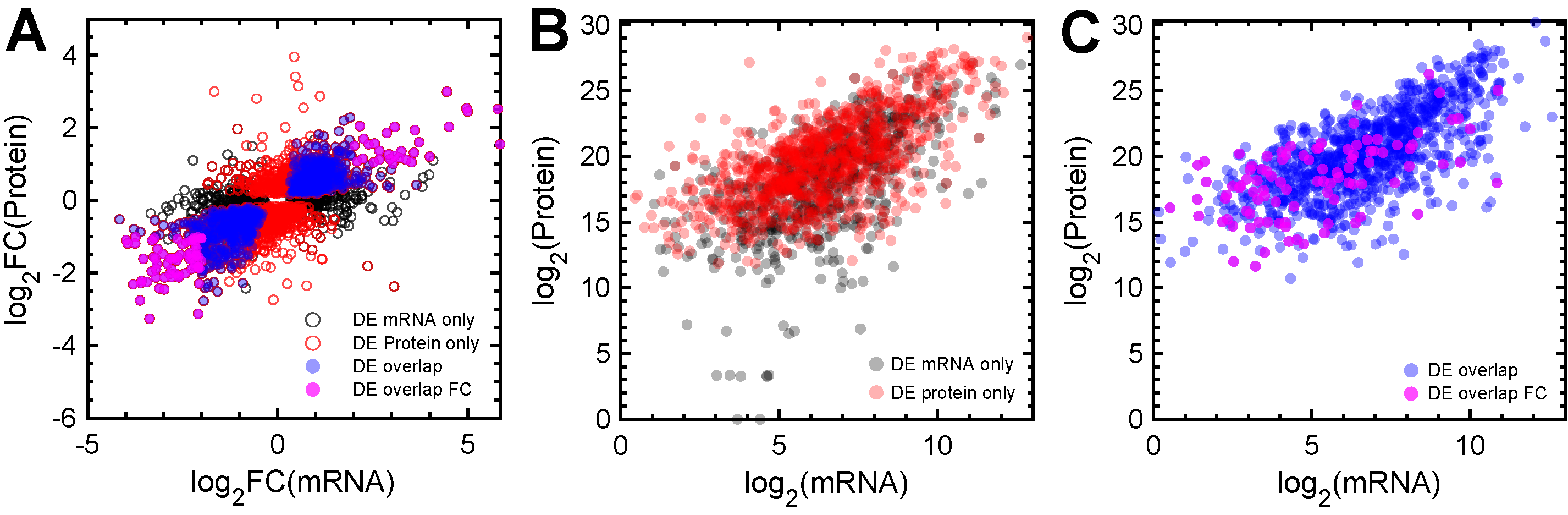

Supplement: FIG S1 [file mbio.03067-22-s0005.tif]

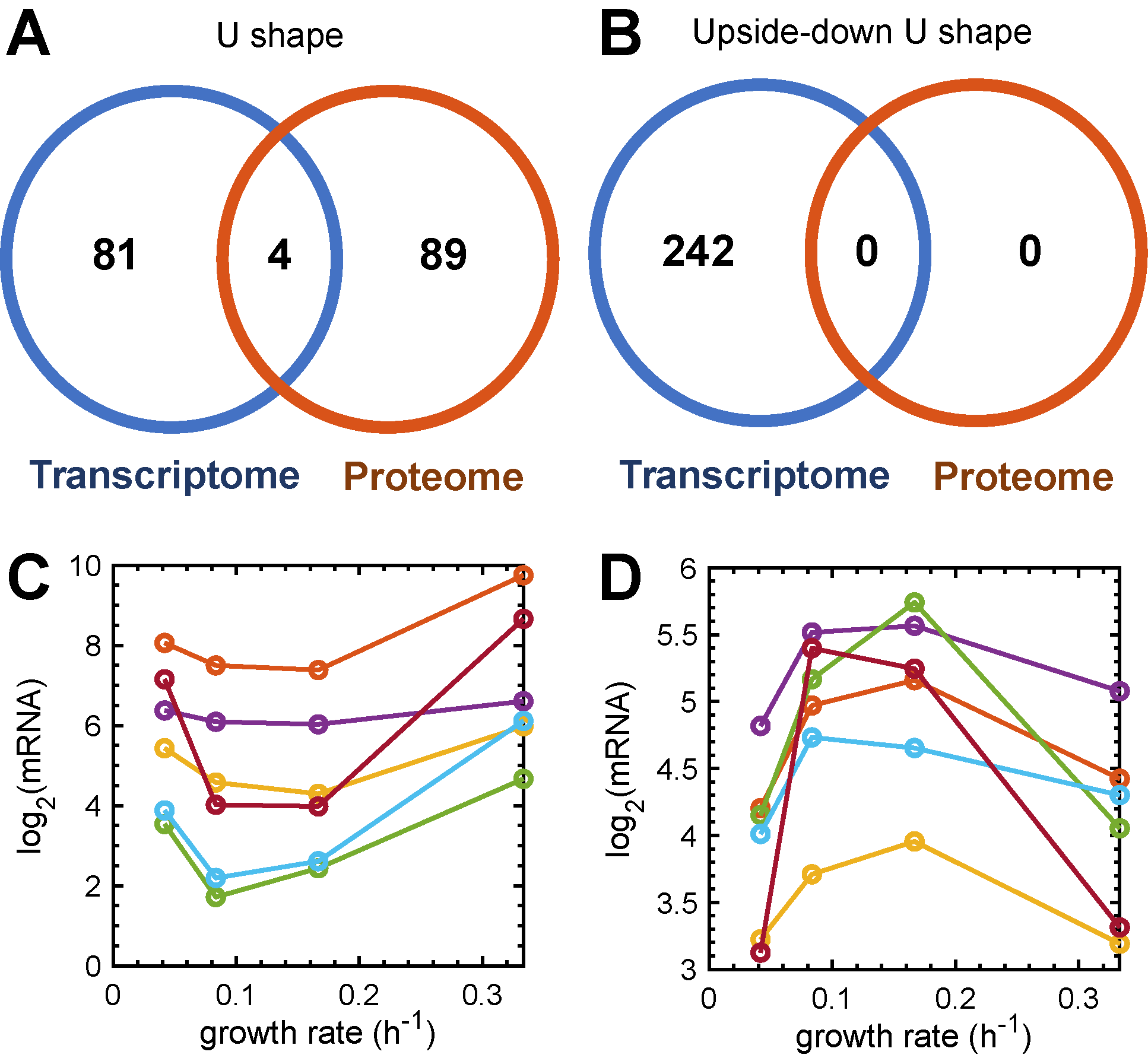

Supplement: FIG S2 [file mbio.03067-22-s0006.tif]

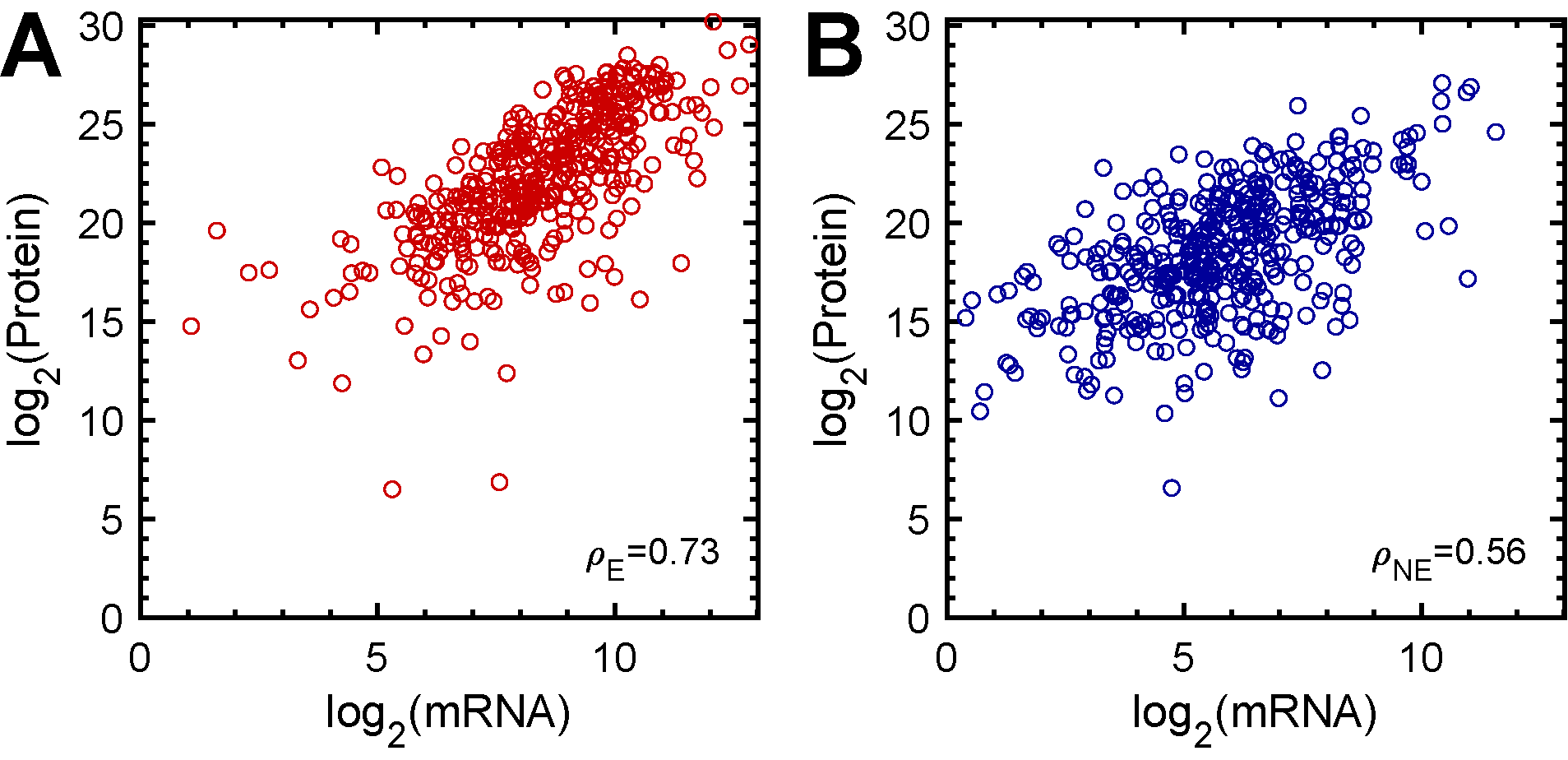

Supplement: FIG S3 [file mbio.03067-22-s0007.tif]

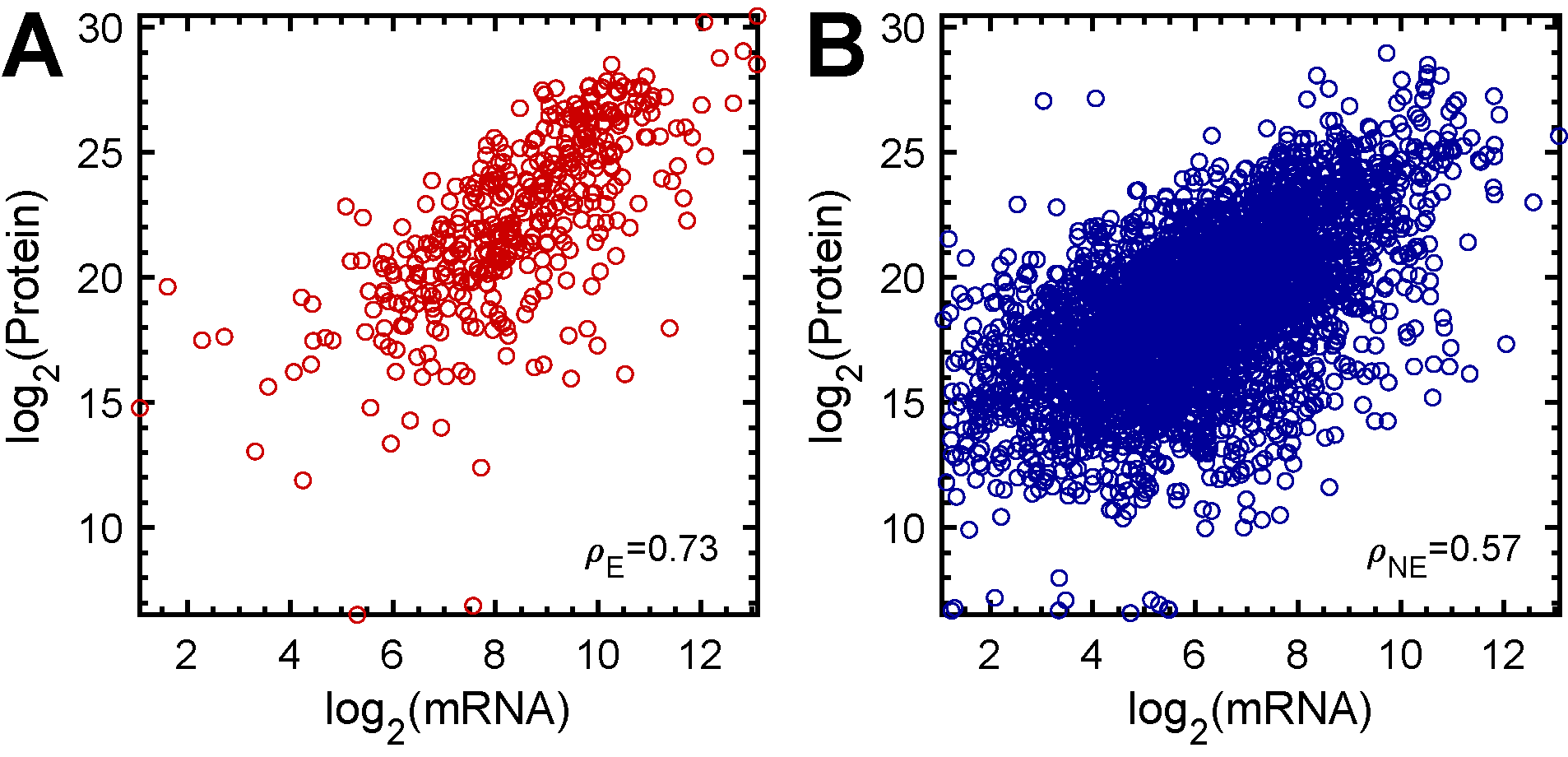

Supplement: FIG S4 [file mbio.03067-22-s0008.tif]

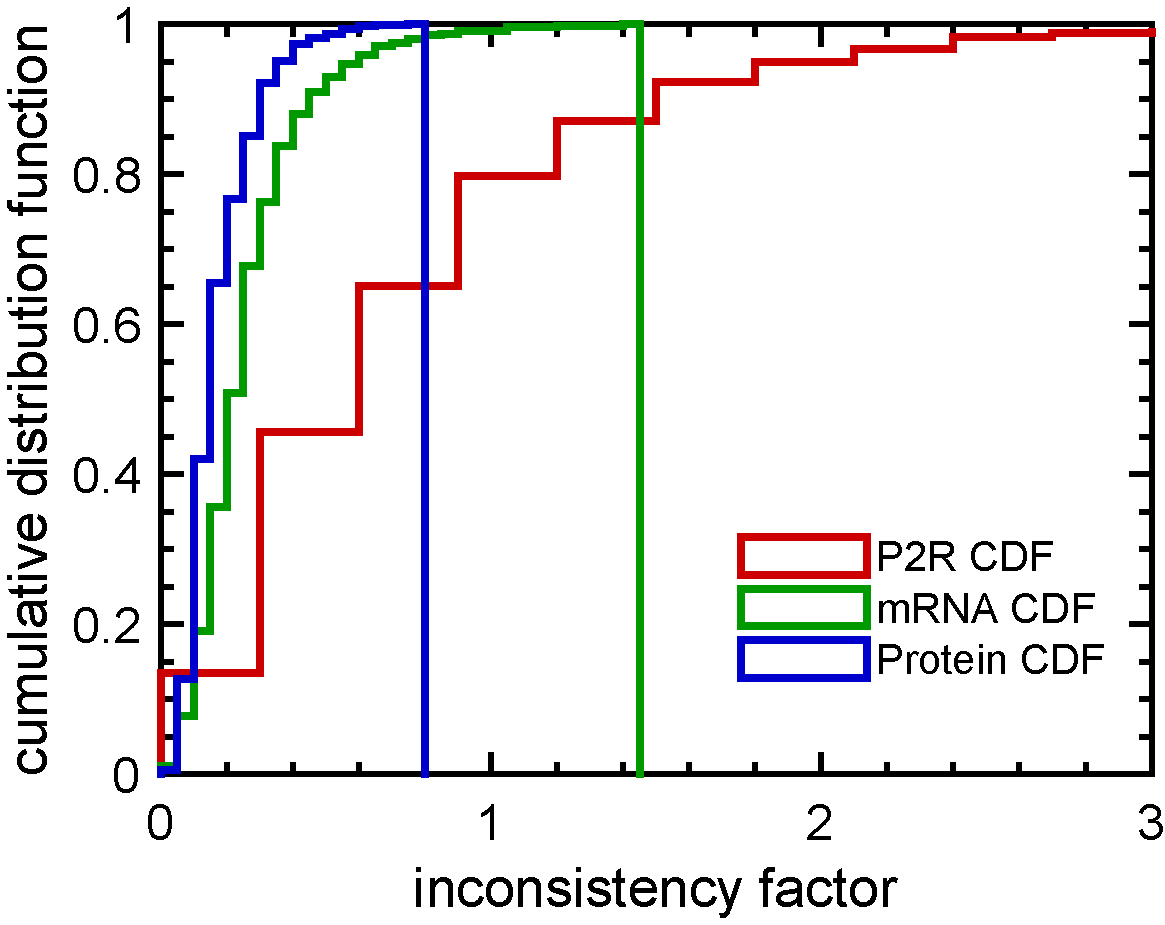

Supplement: FIG S6 [file mbio.03067-22-s0010.tif]

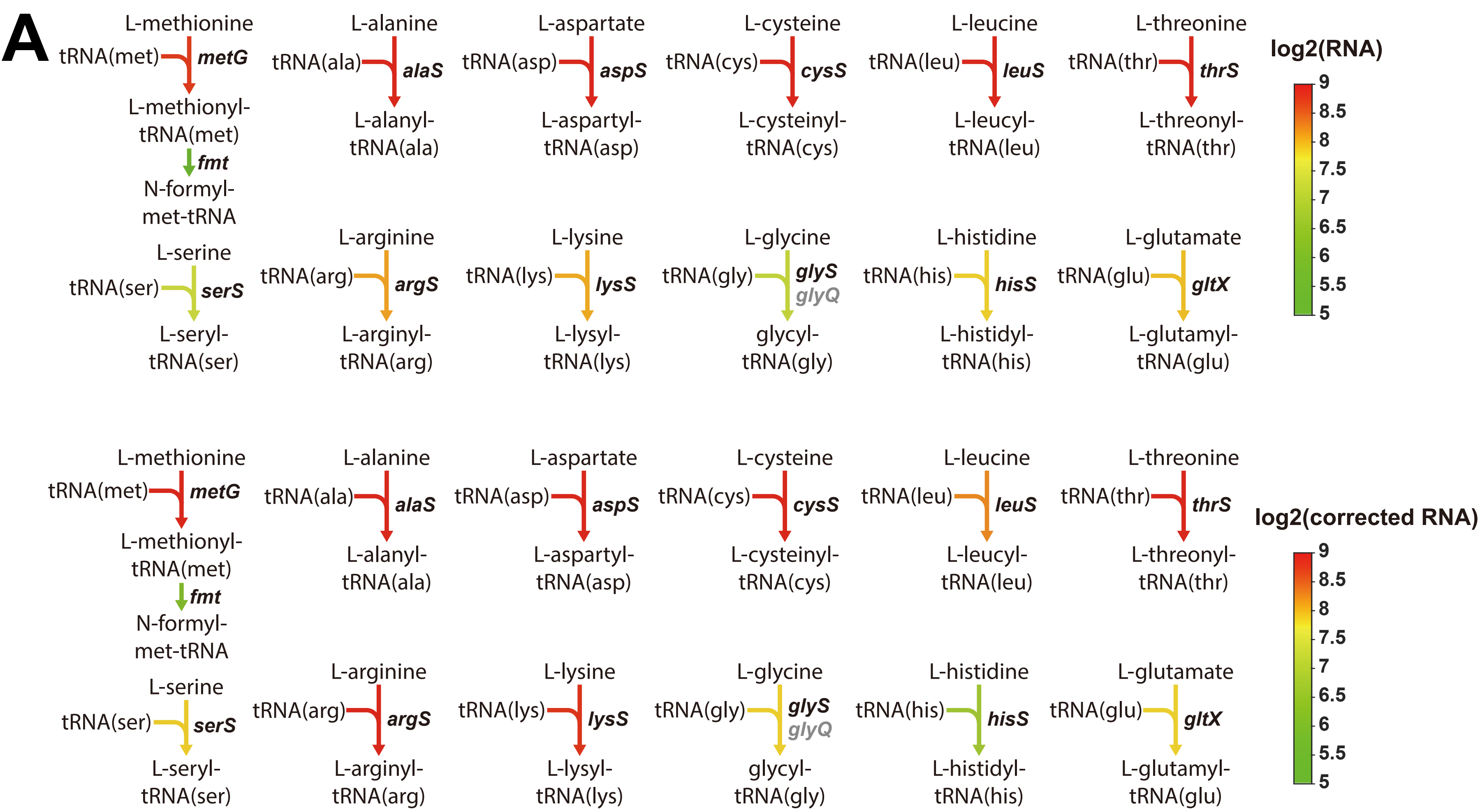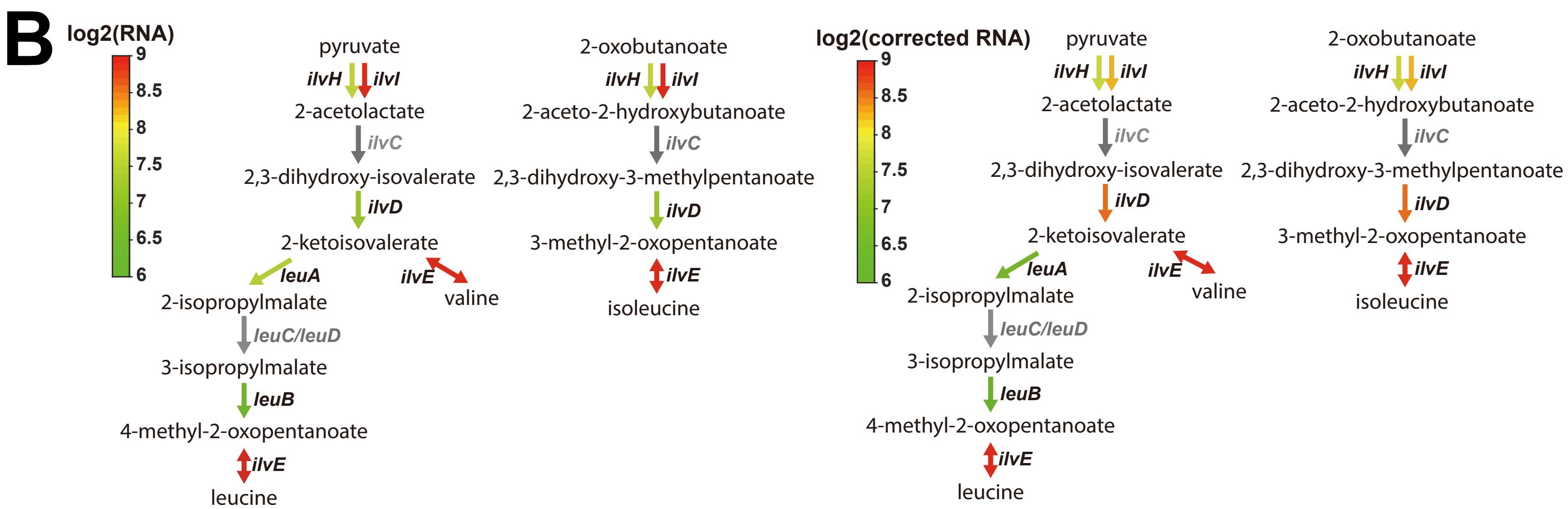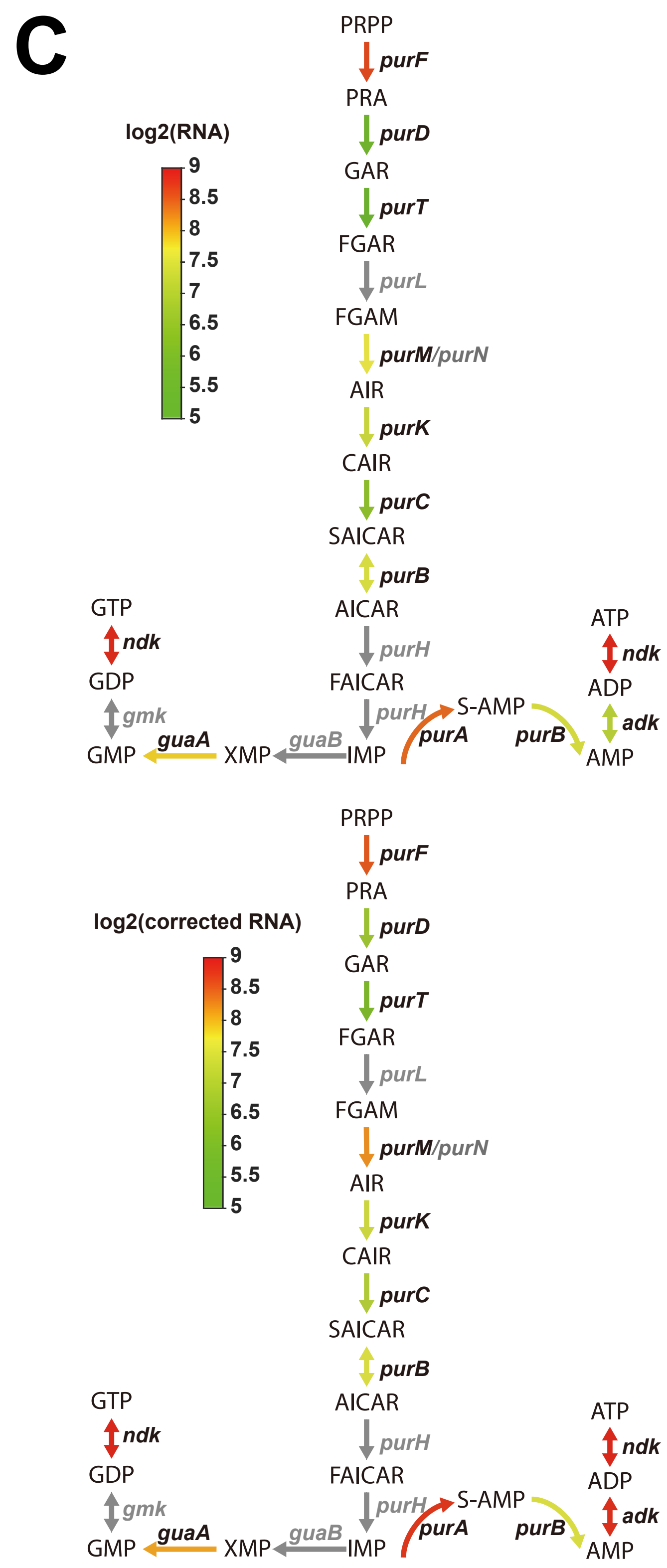

Supplement: FIG S5 [file mbio.03067-22-s0009.pdf]
